# Gene identification, expression analysis, and molecular docking of SAT and OASTL in the metabolic pathway of selenium in *Cardamine hupingshanensis*

**DOI:** 10.1007/s00299-024-03227-6

**Published:** 2024-05-22

**Authors:** Yushan Chen, Yao Li, Guoqiang Luo, Cihang Luo, Zhijing Xiao, Yanke Lu, Zhixin Xiang, Zhi Hou, Qiang Xiao, Yifeng Zhou, Qiaoyu Tang

**Affiliations:** 1 Hubei Key Laboratory of Biological Resources Protection and Utilization, Hubei Minzu University, Enshi, 44500 China; 2 Hubei Key Laboratory of Selenium Resource Research and Biological Application, Hubei Minzu University, Enshi, 44500 China; 3 College of Biological and Food Engineering, Hubei Minzu University, Enshi, 44500 China; 4 College of Forestry and Horticulture, Hubei Minzu University, Enshi, 44500 China

**Keywords:** Serine acetyltransferase, *O*-Acetylserine (thiol) lyase, *Cardamine hupingshanensis*, VIGS, Selenium metabolism

## Abstract

**Key message:**

Identification of selenium stress-responsive expression and molecular docking of serine acetyltransferase (SAT) and *O*-acetyl serine (thiol) lyase (OASTL) in *Cardamine hupingshanensis.*

**Abstract:**

A complex coupled with serine acetyltransferase (SAT) and *O*-acetyl serine (thiol) lyase (OASTL) is the key enzyme that catalyzes selenocysteine (Sec) synthesis in plants. The functions of *SAT* and *OASTL* genes were identified in some plants, but it is still unclear whether *SAT* and *OASTL* are involved in the selenium metabolic pathway in *Cardamine hupingshanensis*. In this study, genome-wide identification and comparative analysis of *ChSAT*s and *ChOASTL*s were performed. The eight *ChSAT* genes were divided into three branches, and the thirteen *ChOASTL* genes were divided into four branches by phylogenetic analysis and sequence alignment, indicating the evolutionary conservation of the gene structure and its association with other plant species. qRT-PCR analysis showed that the *ChSAT* and *ChOASTL* genes were differentially expressed in different tissues under various selenium levels, suggesting their important roles in Sec synthesis. The *ChSAT1;2* and *ChOASTLA1;2* were silenced by the VIGS system to investigate their involvement in selenium metabolites in *C. hupingshanensis*. The findings contribute to understanding the gene functions of *ChSAT*s and *ChOASTL*s in the selenium stress and provide a reference for further exploration of the selenium metabolic pathway in plants.

**Supplementary Information:**

The online version contains supplementary material available at 10.1007/s00299-024-03227-6.

## Introduction

Selenium is among the essential trace elements for mammals and mainly performs its biological functions as selenocysteine to synthesize selenoprotein (Schomburg et al. 2004). In the past century, selenium deficiency was discovered to be the main reason for endemic diseases, such as Keshan disease and Kashin–Beck disease (Jia et al. 2022; Wang et al. 2020). Selenocysteine (Sec) is the 21st amino acid encoded by TGA, which was discovered in 1986 and plays a crucial role in the active center of some antioxidant enzymes, including the glutathione peroxidase (GSH-Px) family and thioredoxin reductase (TrxR). In addition, Sec can effectively remove hydrogen peroxide (H_2_O_2_), lipid peroxides, reactive oxygen species (ROS), and other radicals to protect cells from oxidative damage and maintain the integrity of the plasma membrane (Brigelius-Flohé and Flohé 2020; Chambers et al. 1986; Tindell et al. 2018). In recent years, selenium-containing compounds have been applied to treat cancer and prevent diseases from the immune system (Kuršvietienė et al. 2020; Wallenberg et al. 2014).

Selenium has also been found to be a beneficial element in plants because it affects plant growth and development (Yang et al. 2022). Plants can be divided into selenium non-accumulators, selenium accumulators, and selenium hyperaccumulators based on their selenium content (Szőllősi et al. 2022). In recent years, an increasing number of selenium hyperaccumulator plants have been found, and these plants mainly include *Neptunia amplexicaulis* (legume), *Astragalus disulfide* (legume)*, Stanleya pinnata* (brassicaceae), and *Cardamine hupingshanensis* (brassicaceae) (Wangeline et al. 2011). In addition, the selenium accumulation and tolerance mechanisms have become a research focus (Das and Biswas 2022; Kushwaha et al. 2022). Surprisingly, the selenium content of *N. amplexicaulis*, an herbaceous legume from Australia, has been recorded up to 4334 mg kg^−1^ DW in foliar tissue. MeSeCys and SeMet were mainly metabolites found in *S. pinnata* and *A. disulfide*, which are well-known selenium hyperaccumulators from Brassicaceae and legumes, respectively (Chao et al. 2022; Lima et al. 2022; Sors et al. 2009). They have pharmacological value for dietary supplements due to their high bioavailability (Harvey et al. 2020). SeCys was detected in the leaf-edge globular structures, central leaf, and leaf midrib of *S. pinnata*, comprising 3–9% of the total selenium content (Freeman et al. 2006). *C. hupingshanensis*, a famous selenium hyperaccumulator plant, was first found in the Yutangba Selenium mineral area, Enshi City, Hubei Province, China (Chi et al. 2018). The roots, stems, and leaves of this plant had an average Se content of 2985, 3329, and 2491 mg kg^−1^ DW, with the major Se species being organic, particularly MeSeCys and Sec (Guo et al. 2022). The synthesis of Sec in these plants is thought to be important for their ability to tolerate and accumulate high levels of selenium (Freeman et al. 2010). Sec can be incorporated into specific proteins that are involved in selenium metabolism and detoxification. These selenoproteins may play a role in protecting the plant from the toxic effects of selenium, as well as in sequestering and storing selenium in the plant tissues (Chauhan et al. 2019). Therefore, understanding the synthesis mechanism of Sec is important for further exploration of the selenium metabolism pathways in plants.

The plant *C. hupingshanensis* is usually used as a research object to explore the mechanism of uptake, transport, and metabolism of selenium because the plant exhibits resistance and hyperaccumulation to selenium (Cui et al. 2018). The genome, transcriptome, and proteome of *C. hupingshanensis* were analyzed, the mechanism of selenium resistance and hyperaccumulation was speculated, and the enzymes of the metabolic pathway (Fig. S1) were used to catalyze biochemical reactions, such as reactions with the ATP sulfurylase and selenomethionine cycle enzymes family (Huang et al. 2021; Xiao et al. 2022; Zeng et al. 2024; Zhou et al. 2018). From the schematic, it could be concluded that Sec and SeMet were the junctions of the metabolic pathway of selenium, and these two seleno-amino acids were the main carriers for the physiological function of selenium.

Cysteine synthase, a complex composed of serine acetyltransferase (SAT) and *O*-acetylserine (thiol) lyase (OASTL), is an important component of the intracellular selenium metabolic pathway and plays an important role in the synthesis of Sec (Trippe III and Pilon-Smits 2021). The reaction mechanism of Sec synthesis involves the combination of serine and acetyl-CoA catalyzed by SAT to produce *O*-acetylserine (OAS) and then selenide reaction catalyzed by OASTL to produce Sec (Fig. S2).

The first important member of the cysteine synthase complex (CSC) is serine acetyltransferase (SAT), which catalyzes the synthesis of the intermediate OAS using acetyl-CoA and L-serine (Wirtz and Hell 2006). SAT, as an important regulator of Sec biosynthesis in plants, has also been found in animals, microorganisms, and algae. A total of 5 *SAT* gene members were identified in *Arabidopsis thaliana*, including *AtSAT1*, *AtSAT3*, and *AtSAT5* (in the plasmid, mitochondria, and cytoplasm, respectively) and *AtSAT2* and *AtSAT4* (in the cytoplasm); these genes exhibited lower levels of expression and different protein sequences from the others (Droux 2003; Hell et al. 2002; Kawashima et al. 2005). The SAT monomer consists of an N-terminal α-helix structural domain (α1–α8) and a C-terminal left-parallel β-helix structural domain, which is characteristic of acyltransferases (Johnson et al. 2005). In addition, SATs contain the N-terminal domain structure of serine acetyltransferase (SATase_N, PF06426) and the bacterial transferase hexapeptide (PF00132) as their common motif. The SATase_N motif participates in enzyme activity, and the other motif is crucial for the formation of the CSC (Yeon et al. 2018). SAT not only plays an important role in the synthesis of OAS but also shows resistance to heavy metals such as Ni, Co, Zn, etc., resulting in the accumulation of GSH and increasing antioxidant stress resistance (Hasanuzzaman et al. 2019). In addition, SAT is co-expressed with selenocysteine methyltransferase (SMT), promoting an increase in OAS, as well as the expression of sulfur transporters and the absorption and accumulation of selenium by plants (Özgür et al. 2012).

The other important member of the cysteine synthase complex (CSC) is *O*-acetyl serine (thiol) lyase (OASTL), which catalyzes the synthesis and degradation of Sec using OAS and selenide with pyridoxal 5’-phosphate (PLP) as a cofactor. OASTL has a molecular weight of 60–70 kDa and usually acts in the form of a homodimer, which is ubiquitous in plant and microbial cells (Leustek et al. 2000). The different isoenzymes of OASTL are located in different organelles and play various important functions. Members of the *OASTL* family are involved in the synthesis and degradation of L-Cys and L-Sec, regulating the concentration of intracellular Cys and Sec homeostasis in plants (Assylay et al. 2022). Among the nine *OASTL* family members in *A. thaliana*, the main members of *OASTL* include cytoplasmic *OASTLA1*, chloroplast *OASTLB*, and mitochondrial *OASTLC*, which are involved in cysteine synthesis (Romero et al. 2014). It was shown that in *A. thaliana*, active *OASTLA* and *OASTLB* can improve selenium resistance by degrading L-Cys and L-Sec (Heeg et al. 2008). *AtCYSC1*, *AtCYSD1*, and *AtCYSD2* have very high homology with β-cyano-alanine synthase and scavenge cyanide toxicity, and *AtCS26* and *AtCS-like* synthesize *S*-sulfonylcysteine (Bermúdez et al. 2010; Birke et al. 2012). In addition, OASTL participates in the secondary metabolism of plants and plays an important role in plant growth and development and resistance to abiotic stress (Barroso et al. 1999).

This study aims to identify the members of the *ChSAT* and *ChOASTL* families, perform biological information analysis (including sequence, motif, phylogenetic, domain, and protein modeling), and clarify the physical and chemical properties and basic functions of *ChSAT* and *ChOASTL* genes. In addition, qRT-PCR was used to identify the main gene from the *SAT* and *OASTL* families of *C. hupingshanensis* that reacted to selenite stress, and their functions were verified with the silencing of SAT1;2 and ChOASTLA1;2 using VIGS technology. The molecular docking simulation of OASTL’s affinity to selenium substrates was also implemented to provide a molecular theoretical basis for plant selenium metabolism and Sec biological synthesis.

## Methods

### Identification of ChSAT and ChOASTL genes in *C. hupingshanensis*

The genome and annotation file of *C. hupingshanensis* were obtained from the Genome Warehouse BIG Data Center (https://ngdc.cncb.ac.cn/gwh/) under accession number PRJCA005533. To identify the *SAT* and *OASTL* gene members of *C. hupingshanensis*, we downloaded the nucleotide and protein sequences of the *AtSAT* and *AtOASTL* genes from the *Arabidopsis* Information Resource (https://www.arabidopsis.org/) as reference sequences and searched for the most similar protein sequences of ChSAT and ChOASTL using the blast region function of TBtools software (Chen et al. 2020). To further confirm whether the extracted protein sequences of ChSAT and ChOASTL were correct on the NCBI BLAST (https://blast.ncbi.nlm.nih.gov/Blast.cgi) website, CD-Search (https://www.ncbi.nlm.nih.gov/Structure/cdd/wrpsb.cgi) was used to analyze conserved domains for ChSAT and ChOASTL protein sequences.

### Physicochemical properties and phylogenetic analysis of ChSATs and ChOASTLs

Molecular weight (MW), isoelectric point, and other physicochemical properties of the ChSAT and ChOASTL proteins were analyzed via ExPASy (https://web.expasy.org/protparam). WoLF PSORT (https://wolfpsort.hgc.jp) was subsequently used to predict subcellular localization.

To further understand the evolutionary relationships of the SAT and OASTL families, the SAT and OASTL protein sequences of different plants were downloaded from the NCBI (https://www.ncbi.nlm.nih.gov/) website, with 5 SAT and 9 OASTL from *A. thaliana*, 4 SAT and 9 OASTL from *Brassica napus*, 5 SAT and 6 OASTL from *Glycine max*, 5 SAT and 9 OASTL from *Oryza sativa*, and 4 SAT and 12 OASTL from *Zea mays*. The ChSAT and ChOASTL protein sequences were compared with protein sequences from different plants separately using MEGAX (https://www.megasoftware.net/) software and the evolutionary tree was constructed by the maximum likelihood method.

### Chromosome location and domain analysis of ChSATs and ChOASTLs

Information regarding the location of *ChSAT* and *ChOASTL* genes on the chromosome was obtained by analyzing the gene annotation file of *C. hupingshanensis*, and the gene location visualization (advance) function of TBtools software was used to draw a location map of the gene on the chromosome. Based on the genome annotation file and obtained NCBI CD-Search conserved domain (https://www.ncbi.nlm.nih.gov/Structure/bwrpsb/bwrpsb.cgi) information, we displayed the *ChSAT* and *ChOASTL* gene structure through the Gene Structure View (advance) of TBtools.

Conserved motifs were scanned by the MEME website (http://meme-suite.org/tools/meme), with the MEME motifs set to ten. TBtools draws the intron–exon structure of *ChSAT* and *ChOASTL* based on the information extracted from the GFF file of *C. hupingshanensis*. All the SAT and OASTL protein sequences in *C. hupingshanensis* and *A. thaliana* were aligned by ClustalW (https://www.genome.jp/tools-bin/clustalw), and their alignment results were aligned by ESPript 3.0 (https://espript.ibcp.fr/ESPript/cgi-bin/ESPript.cgi) for further processing to output the image.

### Homology modeling and verification of ChSATs and ChOASTLs

The ChSAT and ChOASTL protein sequences were submitted to SOPMA (https://npsa-prabi.ibcp.fr/cgi-bin/npsa_automat.pl?page=/NPSA/npsa_sopma.html) for secondary structure prediction. In addition, potential templates for different proteins were searched in the SWISS-MODEL template library. Based on the high similarity score, the best crystal structures were selected as templates, and the ChSAT and ChOASTL protein sequences were submitted to the SWISS-MODEL web server (https://swissmodel.expasy.org/) for homology modeling. The protein model quality of the final 3D model of ChSAT and ChOASTL was authenticated by parameters such as GMQE and QMEANDisCo global (Studer et al. 2020).

### Ligand preparation and molecular docking

The structures of the ligand compounds (OAS, hydrogen sulfide, hydrogen selenide, selenophosphate) were obtained from the ChemSpider database (http://www.chemspider.com/), and the 3D structures were plotted with ChemSketch and saved in Protein Data Bank format (Hunter 1997). Prankweb was used to predict protein active sites (Jendele et al. 2019). ChOASTL protein and ligand compound docking experiments were performed using AutoDock v4.2 and AutoDock Vina v1.1.2 to calculate the Gasteiger charge of protein–ligand interactions (Eberhardt et al. 2021).

First, the ChOASTL protein-PLP-OAS ternary complex PDB file was formed by docking the binding pocket of each ChOASTL protein molecule to the cofactor PLP and then docking it to the substrate OAS. Then hydrogen sulfide, hydrogen selenide, and selenophosphate were each docked with the ChOASTL protein-OAS binary complex PDB file vina to form the ChOASTL protein-PLP-OAS-selenophosphate tetrameric complex PDB file. The interactions (hydrogen bonding and hydrophobic interactions) of the ChOASTL protein-OAS-selenophosphate complexes were analyzed and visualized using PILP and PyMol (Adasme et al. 2021; Seeliger and de Groot 2010), and heatmaps of the docking binding energies were generated using TBtools software.

### RNA isolation and gene expression analysis

In our research, the seeds of *C. hupingshanensis* were sourced from the Yutangba Colour Mine in Enshi, Hubei Province, China, and were cultivated in our laboratory after seed collection. The seedlings were cultivated at a temperature of approximately 22 °C and in a pattern of alternating 12 h of light and 12 h of darkness in the plant culture chamber. Thirty-six seedlings of approximately 10 cm in height at the ten-leaf stage were selected. The roots were rinsed to remove the vermiculite and equalized in Hoagland solution for 2 days. The samples were treated with different selenium concentrations (100 µg Se L^−1^, and 80,000 µg Se L^−1^), with the experimental samples untreated with Se as the control group. Leaves and roots of three seedlings were isolated from each treatment at 3, 6, 12, and 24 h, and these samples were then snap-frozen in liquid nitrogen to extract their RNA.

Total RNA was extracted from root and leaf samples using the *TransZol™* Up Plus RNA Kit (TransGen Biotech, China) and NanoDrop 2000 (Thermo Fisher Scientific, USA) to detect RNA concentration, and quality RNA integrity and gDNA contamination were examined by 1.0% agarose gel electrophoresis. RNA from each sample (1 μg) was used to synthesize first-strand cDNA according to the user manual of a HiScript III RT SuperMix for qPCR (+gDNA wiper) kit (Vazyme, China). Real-time qPCR on ABI StepOne Plus (Thermo Fisher Scientific, USA) was performed. The expression of target genes in the samples was detected using the Hieff qPCR SYBR Green Master Mix (High Rox Plus) kit (Yeasen Biotechnology (Shanghai) Co., Ltd., China). Each reaction mixture (10 μL) consisted of 5 μL of Hieff qPCR SYBR Green Master Mix (High Rox Plus), 1 μL of cDNA, 0.4 μL of forward primer (10 μmol L^−1^), 0.4 μL of reverse primer (10 μmol L^−1^), and 4 μL of RNase-free ddH_2_O. The PCR program was as follows: 95 °C for 5 min, 40 cycles of denaturing at 95 °C for 10 s, annealing at 55 °C for 20 s, and then extension at 72 °C for 20 s. Instrument default settings were used during the melting curve stage. Sample cycle threshold (Ct) values were standardized for each template based on the actin gene control primer reaction, and gene expression was calculated using the 2^−ΔΔCT^ method (Livak and Schmittgen 2001). The results were analyzed, and graphical representation was carried out using GraphPad Prism version 9.0.0, GraphPad Software, San Diego, California USA, www.graphpad.comprism. All analyses were performed in triplicate. The primers used for the qRT-PCR analysis of *ChSAT* and *ChOASTL* are listed in Table S2.

### Virus-induced gene silencing (VIGS) of ChSAT1;2 and ChOASTLA1;2 genes, and analysis of selenium metabolites in *C. hupingshanensis*

The experimental methodology was referenced from the previous publications (Li et al. 2023; Robertson 2004; Rössner et al. 2022). TRV1 and TRV2 were used to construct VIGS vectors, and the *ChSAT1;2* (350 bp) and *ChOASTLA1;2* (350 bp) fragments were cloned into the TRV2 vector after *Eco*RI and *Xho*I digests. The recombinant vector and pTRV1 were transferred into *Agrobacterium tumefaciens* GV3101 chemically competent cell, which was grown at 28 °C for 2 days in Luria–Bertani medium (50 μg mL^−1^ rifampicin, 50 μg mL^−1^ kanamycin). The cells were centrifuged at 5000 rpm for 5 min at 4 °C, and the collected bacteria were resuspended with resuspension solution (150 μM acetosyringone, 10 mM MES, and 10 mM MgCl_2_) to a final OD_600_ = 1.0. The resuspended cells were incubated at 100 rpm for 2 h at 28 °C. *Agrobacterium tumefaciens* carrying the pTRV1 vector was mixed with pTRV2, pTRV-*ChSAT1;2* and pTRV-*ChOASTLA1;2* in a 1:1 mixture before infestation, and pTRV1 and pTRV2 co-infested quilt was used as a negative control. The four-leaf stage of the seedlings of *C. hupingshanensis* was used for the VIGS experiment, and the above infected solution was injected into each leaf, followed by a 24-h light avoidance treatment. After injection, the seedlings were cultured in a plant culture chamber with a temperature of about 22 °C, 12 h of light and 12 h of darkness alternating. After 3 weeks of silencing treatment, gene expression of the infected plants was detected and the silencing plants were then treated with selenium (100 µg Se L^−1^) for a continuous treatment of 24 h. The leaves of each plant were immediately frozen in liquid nitrogen and stored at −80 °C for detection and analysis of selenium metabolite content. All analyses were performed in triplicate. The primers used for the VIGS analysis of pTRV-*ChSAT1;2* and pTRV-*ChOASTLA1;2* are listed in Table S2.

To analyze the content of the five selenium metabolites, 0.05 g of the samples were crushed and added with Tris–HCl extraction solution (containing Proteinase K and Proteinase E), shaken at 37 °C overnight, and then centrifuged at 5000 rpm for 10 min, and the supernatant was passed through 0.22 μm filtration membrane and then left to be measured. Three replicates were made for each sample, and the blank control was made at the same time. The samples were analyzed by high-performance liquid chromatography–inductively coupled plasma mass spectrometry (HPLC–ICP-MS). The HPLC system consisted of the 1260 Infinity II Prime Liquid Chromatograph and the separation was performed using Agilent ZORBAX SB-A Chromatography Columns (4.6 × 250 mm, 5 μm diameter). The mobile phase consisted of 20 mM citric acid, 5 mM sodium hexanesulfonate, and pH 4.5, which was pumped through the analytical column at a flow rate of 1.0 mL min^−1^. An 8800 triple tandem inductively coupled plasma mass spectrometer (Agilent, USA) was used as the selenium-specific detector. The ICP-MS system was operated under the following conditions: RF power of 1550 W, RF voltage of 1.8 V, sampling depth of 8 mm, carrier gas flow rate of 1 L min^−1^, acquisition mode of TRA, and elemental detection of ^78^Se. The analytical standard substances, including Se (IV) (GBW10032), Se (VI) (GBW10033), MeSeCys (GBW10088), SeMet (GBW10034), and SeCys_2_ (GBW10087), were obtained from the National Research Centre for Accredited Standard Substances (Beijing, China), and the above standards were prepared to a concentration of 1.0 mg L^−1^ in ultrapure water and stored at 4 °C. All the solutions were prepared and the samples were processed with Milli-Q water (18.2 MΩ cm). The peak areas were determined using the data analysis program of Agilent ICP-MS Mass Hunter software. The selenium compounds were quantified using external standard curves established for each of the five compounds.

## Results

### Identification and analysis of SAT and OASTL genes in *C. hupingshanensis*

Eight members of the *ChSAT* gene family were found in the genome of *C. hupingshanensis* (the Genome Warehouse BIG Data Center accession number PRJCA005533) by comparison with the genome sequences of *A. thaliana*. The gene names of *ChSAT* family members were determined by sequence homology of *ChSAT*. Detailed information about the members of the *ChSAT* gene family, including the number of amino acid residues in the protein, theoretical isoelectric point, molecular weight, and other related physicochemical property indices, is given in Table 1. The amino acid residues range from 262 to 398, the molecular weight ranges from 27.95 to 52.23 kDa, and the isoelectric point values range from 5.11 to 8.35 of the ChSAT family. Most ChSAT proteins are in the faintly acidic or neutral cellular environment in *C. hupingshanensis* except for ChSAT4. The members of the ChSAT protein family, in addition to ChSAT1;1 and ChSAT1;2, are presumed to be stable proteins by the prediction of instability coefficients. The results of subcellular localization analysis showed that proteins of the ChSAT were distributed in the cytoplasm except for the ChSAT3 protein, which was located in the mitochondrion.

**Table 1:** Identification of basic physicochemical properties of *ChSAT* gene family members

Gene ID	Gene name	Amino acid (aa)	Molecular weight (kDa)	Isoelectric point (pI)	Instability index	Subcellular localization
Chu003685	ChSAT1;1	312	34.22	7.18	45.33	Cytoplasm
Chu013066	ChSAT1;2	312	34.11	7.16	41.65	Cytoplasm
Chu020773	ChSAT2;1	262	27.95	6.49	30.35	Cytoplasm
Chu050052	ChSAT2;2	323	34.78	5.78	36.51	Cytoplasm
Chu049653	ChSAT3	398	42.68	5.11	39.01	Mitochondrion
Chu026957	ChSAT4	393	52.23	8.35	38.85	Cytoplasm
Chu011818	ChSAT5;1	309	32.46	6.92	38.44	Cytoplasm
Chu040697	ChSAT5;2	311	32.64	6.89	39.27	Cytoplasm

A total of 13 members of the *OASTL* gene family were characterized in the *C. hupingshanensis* genome by homology searches. The ChOASTL proteins ranged from 322 to 430 amino acid residues, molecular weights from 32.01 to 45.96 kDa, and isoelectric point values from 5.29 to 8.77 (Table 2). A pI of ChOASTL lower than 7.0 would be similar to the cytoplasmic pI, in contrast to the chloroplast and mitochondrial pI. Similar phenomena were observed in *A. thaliana* and *Sorghum bicolor* (Akbudak et al. 2019). These pI values may indicate the location of OASTL proteins in the cell. The members of the ChOASTL protein family, in addition to ChOASTLB1, ChOASTLB2, and ChOASTLC1, are presumed to be stable proteins by the prediction of instability coefficients. The coding sequences and protein sequences of *ChSAT* and *ChOASTL* genes are shown in Table S1.

**Table 2:** Identification of basic physicochemical properties of *ChOASTL* gene family members

Gene ID	Gene name	Amino acid (aa)	Molecular weight (kDa)	Isoelectric point (pI)	Instability index	Subcellular localization
Chu019397	ChOASTLA1;1	322	33.89	5.9	27.5	Cytoplasm
Chu029016	ChOASTLA1;2	322	34.11	5.9	29.41	Cytoplasm
Chu048695	ChOASTLA2	342	36.45	8.25	31.37	Chloroplast
Chu035096	ChOASTLB1	394	41.94	8.38	42.31	Chloroplast
Chu007319	ChOASTLB2	394	41.91	8.68	44.75	Chloroplast
Chu046136	Ch*OASTLC1*	430	45.96	8.78	42.19	Chloroplast
Chu025039	Ch*OASTLC2*	429	45.82	8.73	39.53	Chloroplast
Chu045982	ChCYS*C1;1*	368	39.76	8.77	29.95	Mitochondrion
Chu026205	ChCYS*C1;2*	368	39.9	8.11	29.63	Mitochondrion
Chu037773	ChCYS*D2;1*	323	34.36	5.65	31.13	Cytoplasm
Chu044352	ChCYS*D2;2*	338	36.59	5.71	36.49	Cytoplasm
Chu023729	Ch*CS26*	405	43.58	7.75	39.09	Chloroplast
Chu037774	Ch*CS-like*	302	32.01	5.29	25.28	Chloroplast

### Chromosomal localization and domain analysis of SATs and OASTLs in *C. hupingshanensis*

**Fig. 1:**
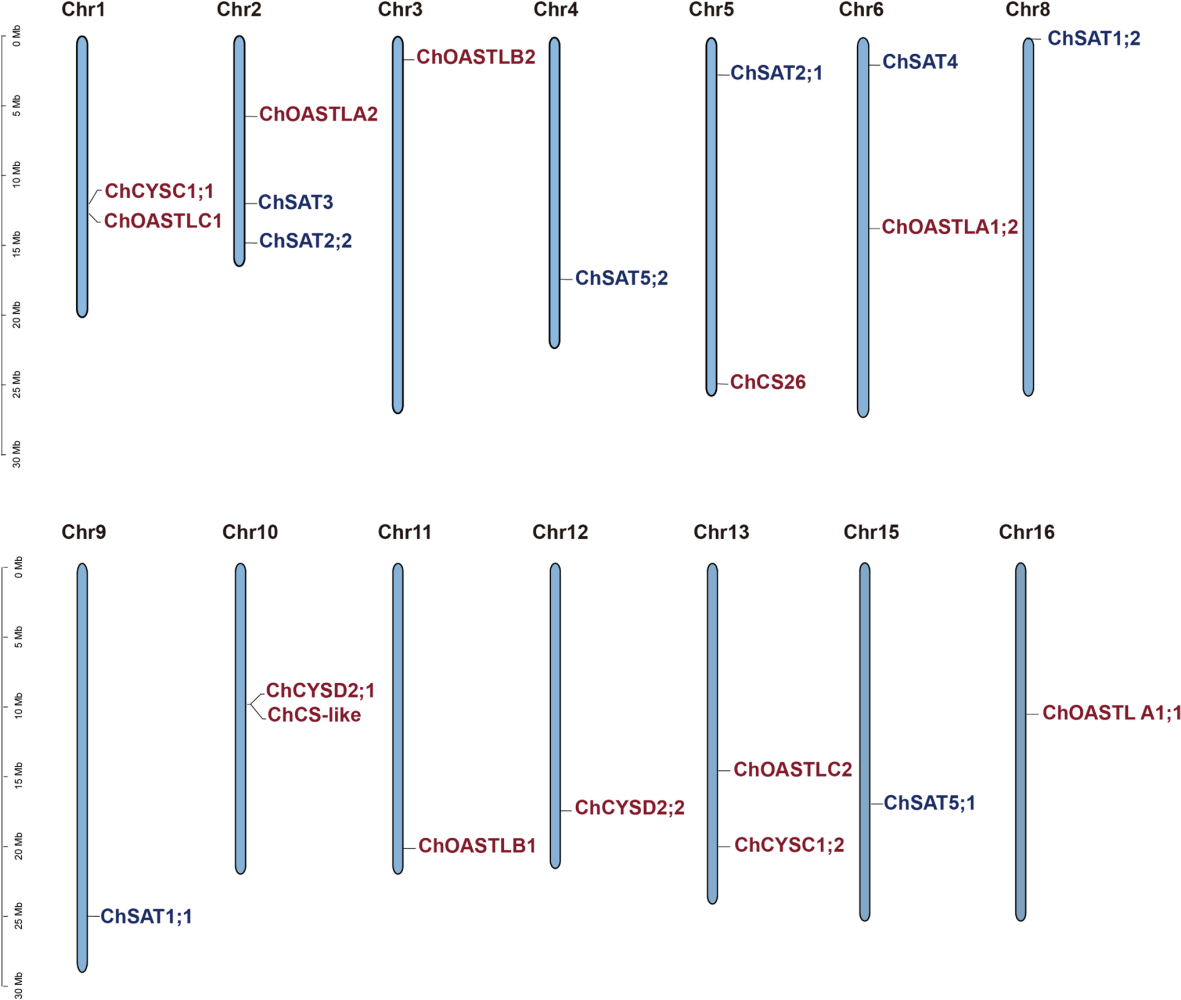
Chromosomal localization of the *SAT* and *OASTL* genes in *C. hupingshanensis. ChSAT*s are marked in red and *ChOASTL*s are marked in *blue* (color figure online)

The distribution of *ChSAT* and *ChOASTL* genes on the 16 chromosomes of *C. hupingshanensis* is relatively scattered (Fig. 1). Except for chromosome 2, which contains three genes (*ChOASTLA2*, *ChSAT2*;*2* and *ChSAT3*), the chromosomes of *C. hupingshanensis* contain one or two *ChSAT* and *ChOASTL* genes. A close linkage was found between *ChCYSD2*;*1* and *ChCS-like*, which are in the middle of chromosome 10, indicating that members of the *ChOASTL* gene family have experienced tandem repeats during evolution.

**Fig. 2:**
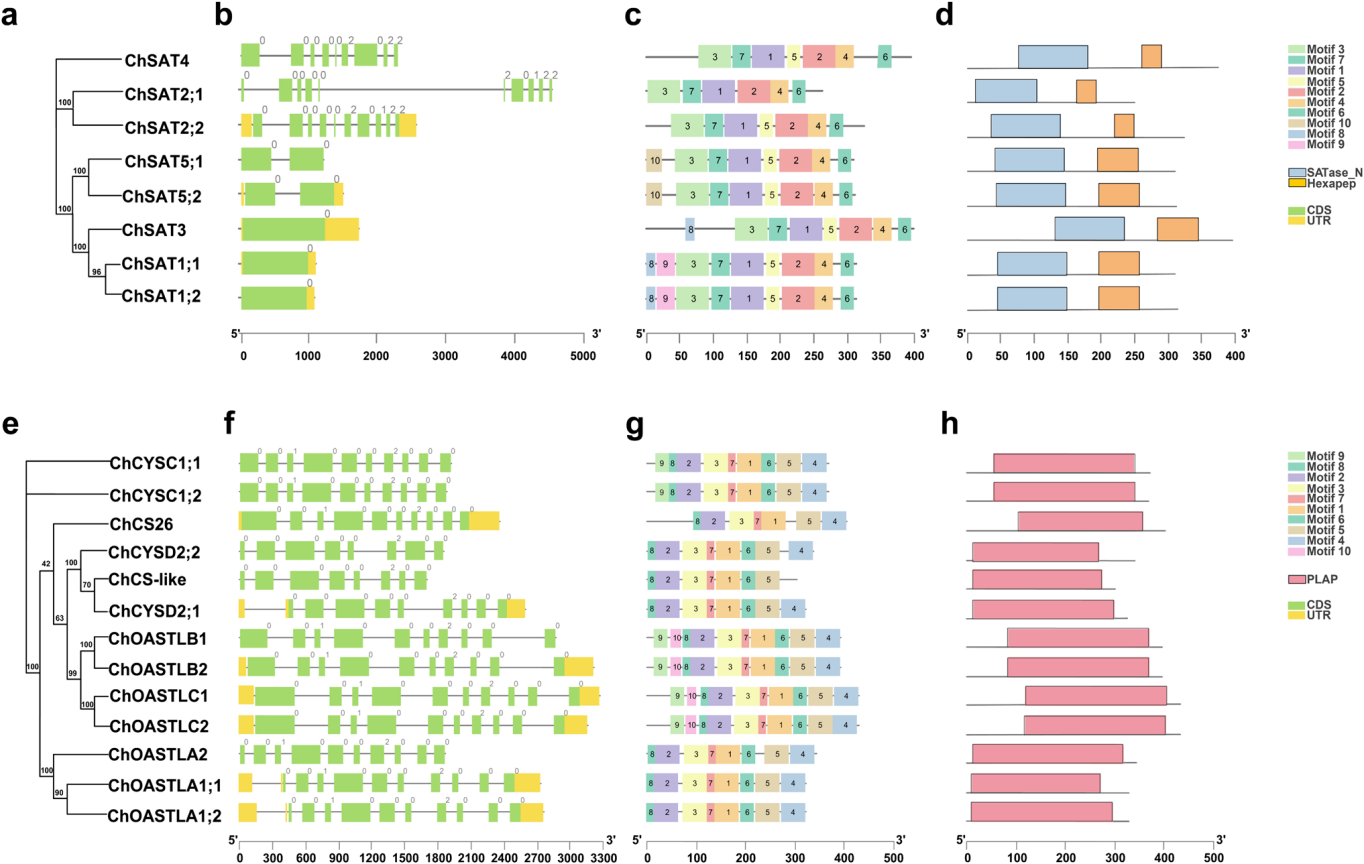
Phylogenetic tree, gene structure, conserved motifs, and structural domains of *ChSAT* and *ChOASTL* genes. **a** The phylogenetic tree of ChSAT proteins. **b** Intron and exon structures of *ChSAT* genes; exons and introns are represented by *green squares* and *black lines*. **c**, **d** The conserved motifs and structural domains of ChSAT proteins; different conserved motifs and structural domains are marked by *different colors*. **e** The phylogenetic tree of ChOASTL proteins. **f** Intron and exon structures of *ChOASTL* genes; exons and introns are represented by *green squares* and *black lines*. **g**, **h** The conserved motifs and structural domains of ChOASTL proteins; different conserved motifs and structural domains are marked by *different colors* (color figure online)

A simple maximum likelihood phylogenetic tree was constructed for the protein sequences of SAT and OASTL of *C. hupingshanensis* to adequately identify the protein motifs, conserved structural domains, and gene structures (Fig. 2a and e). The gene structures of the coding sequences and untranslated regions (UTRs) of the *SAT* and *OASTL* genes in *C. hupingshanensis* were visualized using TBtools software. The results revealed that the homologous genes from different groups had similar intron or exon numbers and distributions. These results demonstrate that the same subclade of *ChSAT* and *ChOASTL* genes was functionally and structurally conserved, and there exist differences between the different subclades (Fig. 2b and f).

The online software MEME was used to search the conserved motifs of ChSATs and ChOASTLs, and ten conserved motifs with diverse architecture for the ChSATs and ChOASTLs protein sequences were found (Fig. 2c and g). The results indicated that motifs 1–7 predominantly occurred in most ChSATs, whereas motif 5 was not frequently found in ChSAT2;1. In addition, motif 10 is distinctive to ChSAT5;1 and ChSAT5;2, and motif 9 is also characteristic of ChSAT1;1 and ChSAT1;2. There are eight mainly conserved motifs (motifs 1, 2, 3, 4, 5, 6, 7, and 8) found in the ChOASTL protein sequences. But motif 6 and motif 4 were not present in ChCS26, and motif 4 was not present in ChCS-like. Moreover, motif 8 was characteristic of the four members of the ChOASTL family, including the ChOASTLB1, ChOASTLB2, ChOASTLC1, and ChOASTLC2 proteins, which might explain the benign variation in the ChSAT and ChOASTL families during evolution.

The analysis of ChSAT domains using the NCBI CD search server revealed that all proteins contained the conserved serine acetyltransferase N-terminal domain structure (SATase_N, PF06426) and the bacterial transferase hexapeptide (PF00132) domain. In addition, a conserved pyridoxal phosphate-dependent enzyme domain (PF00291, PALP) was detected in all ChOASTL proteins, which could indicate that these enzymes use pyridoxal monohydrate 5’-phosphate (PLP) as a cofactor (Fig. 2d and h).

### Phylogenetic analysis of SATs and OASTLs in *C. hupingshanensis*

**Fig. 3:**
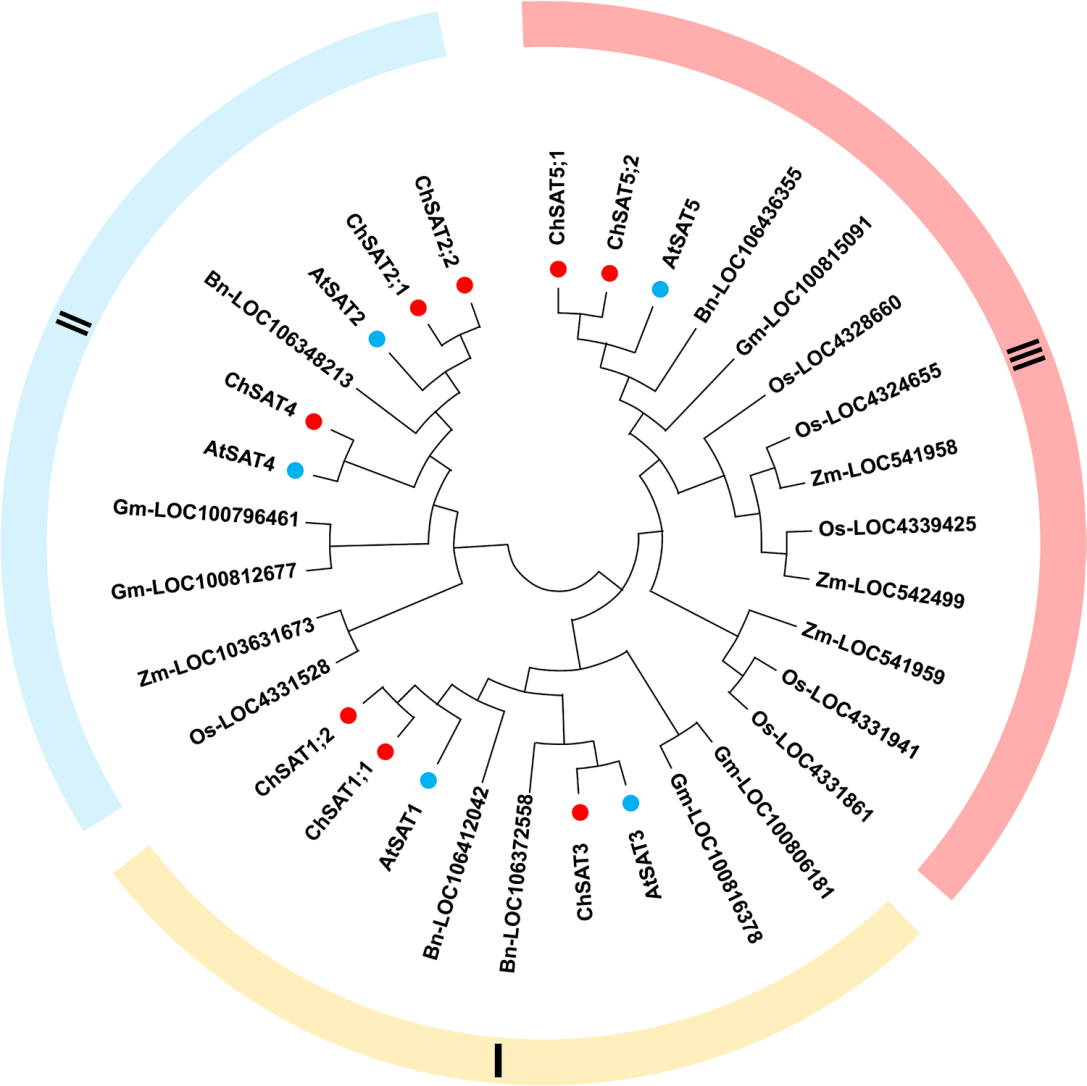
Phylogenetic analysis of the ChSAT families in *C. hupingshanensis*. At: *Arabidopsis thaliana*; Bn: *Brassica napus*; Ch: *Cardamine hupingshanensis*; Gm: *Glycine max*; Os: *Oryza sativa*; Zm: *Zea mays*

To characterize the phylogenetic relationships of *SATs* and *OASTLs* in *C. hupingshanensis* and different plant species and predict their function, we performed phylogenetic trees construction of all *SAT* and *OASTL* genes for six plant species (*C. hupingshanensis*, *A. thaliana*, *Brassica napus*, *Glycine max*, *Oryza sativa*, *Zea mays*). Based on the results of phylogenetic analysis, eight *ChSAT* genes were divided into Groups I–III. *ChSAT1;1*, *ChSAT1;2*, and *ChSAT3* were aggregated in Group I, *ChSAT2;1*, *ChSAT2;2*, and *ChSAT4* were grouped in Group II, and Group III contained *ChSAT5;1* and *ChSAT5;2* (Fig. 3). The same analysis was used for the *OASTL*s. The results showed that all the OASTL genes were classified into four different groups (A, B, C, and D). Groups B, C, and D each have 18, 16, and 14 members, respectively. Group A has ten members, which are present as three *OASTL*s in *C. hupingshanensis* (Fig. S3)*.* The *ChSAT*s and *ChOASTL*s were more closely related to the *AtSAT*s and *AtOASTL*s, possibly because both *C. hupingshanensis* and *A. thaliana* are dicotyledonous Brassicaceae plants, which exhibit evolutionarily close kinship. Based on the comprehensive phylogenetic analysis, the *SAT* and *OASTL* genes were present in every subgroup of monocotyledonous and dicotyledonous plants; that is, the *SAT* and *OASTL* families appeared before the differentiation of monocotyledonous and dicotyledonous plants.

### Functional characteristics analysis of ChSAT and ChOASTL proteins

**Fig. 4:**
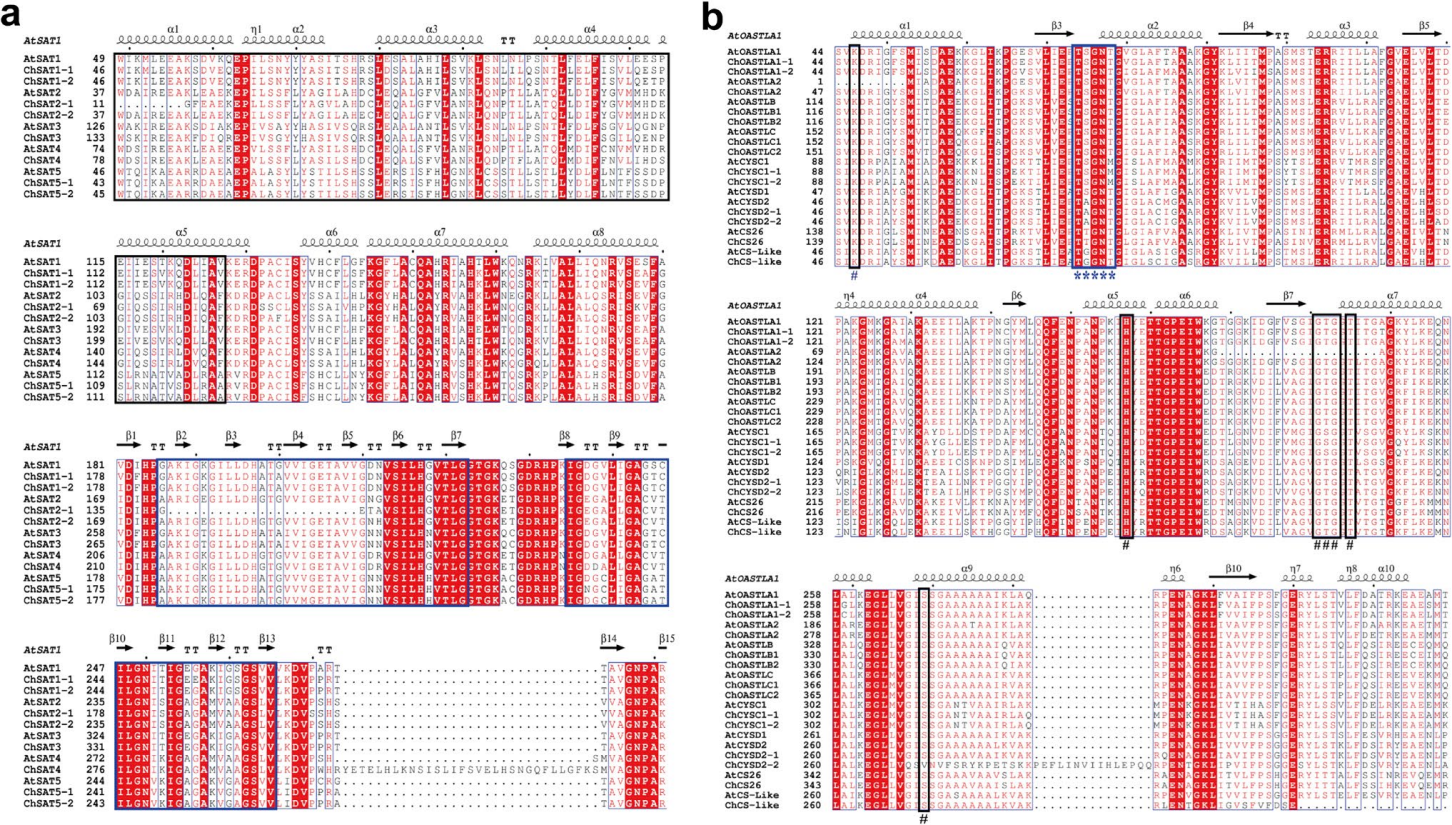
Multiplexed alignment of partial sequences of SAT and OASTL protein in *C. hupingshanensis*. **a** Multiplexed alignment of partial sequences of ChSAT protein. Amino acid residues forming the structural domain of the serine acetyltransferase N-terminal domain structure (SATase_N, PF06426) are marked by *black boxed underlines*, and those of the bacterial transferase hexapeptide domain structure (PF00132) are marked by *blue box underlines*. **b** Multiplexed alignment of the partial sequences of ChOASTL protein. Amino acid residues bound to PLP are marked by *black box lines* and “#”, and those bound to substrate constant are indicated by *blue box lines* and “*” (color figure online)

The alignment of protein sequences in SAT and OASTL processed by ClusterW software showed that the conserved serine acetyltransferase N-terminal domain structure (SATase_N, PF06426) was located in the range of ^49^W–V^128^, while the bacterial transferase hexapeptide domain structure (hexapeptide, PF00132) was located in the range of ^200^G–G^222^ and ^235^I–V^265^ (Fig. 4a, Fig. S4). SATase_N and hexapeptide are common domains in most SATs that are characteristic of the enzyme. However, a conserved motif devoid of 23 residues was also found in the N-terminus of the hexapeptide of ChSAT2;1. Previous research has shown that the highly conserved β1c–β2c loop, which we defined as the β7–β8 loop, plays a key role in the binding of SAT proteins to serine (Yi et al. 2013). In addition, the ten residues of the C-terminus of SAT are used to combine the active site of OASTL and form the cysteine synthase complex (Bogdanova and Hell 1997; Jez and Dey 2013; Ma et al. 2023).

According to the conserved motif analysis of OASTL proteins, the highly conserved PLP binding site was present in all ChOASTLs and consisted of the amino acid residues Lys^46^, His^157^, Gly^181^, Thr^182^, Gly^183^, Thr^185^, and Ser^269^ (Fig. 4b, Fig. S5). Furthermore, binding energy originates from the hydrogen bond between the cofactor and the enzyme(Bonner et al. 2005). Because the PLP binding motif is absent, AtOASTLA2 was considered a nonfunctional pseudogene (Jost et al. 2000). The substrate-binding site of OASTLs also contained another important motif (^74^TSGNT^78^ loop), which was highly conserved in the three major isoforms of the OASTL family, including OASTLA, OASTLB, and OASTLC. The Ser residue of the loop was substituted by Aln in AtCYSD2, ChCYSD2;1 and ChCYSD2;2, by Thr in AtCS26 and ChCS26, and by Gly in AtCS-like and ChCS-like. The two key amino acids Thr^74^ and Ser^75^ of the ^74^TSGNT^78^ loop combine with the sulfide or selenide of the substrate, and this site mutation leads to a reduction in the efficiency of cysteine and selenocysteine synthesis (Kopriva et al. 2009).

### Secondary and tertiary structure prediction of SAT and OASTL proteins in *C. hupingshanensis*

Secondary structures analysis of ChSATs and ChOASTLs using SOPMA online outlets showed that they were enriched in the alpha helix (33.76–47.13%), extended strand (16.50–24.43%), random coil (27.56–43.22%) and contained small amounts of beta turn (6.28–11.99%), indicating some variability in protein structures on ChSATs and ChOASTLs (Table 3). To better visualize the tertiary structure, ChSAT and ChOASTL protein sequences were submitted to the SWISS-MODEL online server and blast-searched for the protein template with the best match. The serine acetyltransferase (SMTL ID: 4n69.1. A) of soybean was used as the template for crystal structures in the homology modeling of SATs, and the predicted tertiary structures of AtSAT and ChSAT are shown in Fig. S6a. Similarly, the crystal structure of the *A. thaliana O*-acetyl serine (thiol) enzyme (SMTL ID: 4aec.1. A) was chosen as the template for homology modeling of OASTLs, and the predicted tertiary structures of AtOASTL and ChOASTL are shown in Fig. S6b. The quality of the 3D structural models of ChSATs and ChOASTLs predicted by SWISS-MODEL used metrics of sequence identity, coverage, and global model evaluation, and the results are presented in Table 3. The sequence identity of the ChSAT and ChOASTL protein models was above 55.00%, and the coverage reached above 0.66. The global model quality evaluation (GMQE) and QMEANDisCo global give an overall quality measure between 0 and 1, with larger numbers indicating a higher expected quality. The valuation results of the quality of the ChSAT and ChOASTL 3D structures showed that the quality of the models provided by SWISS-MODEL met our expectations and could be used in the next study.

**Table 3:** Quality parameters of ChSAT and ChOASTL protein models and secondary structure analysis

Gene	Template	Sequence identity	Coverage	GMQE	QMEAN	Alpha helix	Beta turn	Extended strand	Random coli
ChSAT1;1	4n69.1. A	60.07%	0.89	0.74	0.84	39.10%	7.05%	18.91%	34.94%
ChSAT1;2	4n69.1. A	61.09%	0.88	0.74	0.84	42.95%	8.01%	21.47%	27.56%
ChSAT2;1	4n69.1. A	55.94%	0.77	0.65	0.79	47.13%	6.90%	17.62%	28.35%
ChSAT2;2	4n69.1. A	55.94%	0.73	0.63	0.83	35.29%	8.67%	19.81%	36.22%
ChSAT3	4n69.1. A	60.36%	0.70	0.56	0.82	33.92%	6.28%	16.58%	43.22%
ChSAT4	4n69.1. A	55.00%	0.66	0.51	0.73	37.66%	7.89%	24.43%	30.03%
ChSAT5;1	4n69.1. A	86.23%	0.89	0.79	0.89	44.34%	7.44%	17.80%	30.42%
ChSAT5;2	4n69.1. A	86.59%	0.89	0.78	0.89	45.43%	7.72%	18.33%	28.06%
ChOASTLA1;1	4aec.1. A	72.50%	0.98	0.91	0.88	40.37%	10.87%	18.94%	29.81%
ChOASTLA1;2	4aec.1. A	69.30%	0.98	0.90	0.88	40.37%	10.56%	17.70%	31.37%
ChOASTLA2	4aec.1. A	68.34%	0.93	0.83	0.82	37.72%	11.99%	19.30%	30.99%
ChOASTLB1	4aec.1. A	82.34%	0.98	0.83	0.86	35.79%	9.14%	17.51%	37.56%
ChOASTLB2	4aec.1. A	82.99%	0.87	0.79	0.86	33.76%	8.88%	16.50%	40.86%
ChOASTLC1	4aec.1. A	92.25%	0.99	0.79	0.87	38.37%	9.77%	18.37%	33.49%
ChOASTLC2	4aec.1. A	92.72%	0.99	0.79	0.86	41.03%	7.93%	17.25%	33.80%
ChCYSC1;1	4aec.1. A	60.69%	0.86	0.78	0.86	38.59%	9.78%	16.58%	35.05%
ChCYSC1;2	4aec.1. A	60.38%	0.86	0.78	0.85	37.50%	9.78%	18.21%	34.51%
ChCYSD2;1	4aec.1. A	67.29%	0.99	0.89	0.85	39.94%	10.84%	19.81%	29.41%
ChCYSD2;2	4aec.1. A	61.37%	0.95	0.80	0.79	37.87%	11.24%	20.71%	30.18%
ChCS26	4aec.1. A	63.06%	0.78	0.67	0.82	35.06%	10.37%	20.00%	34.57%
ChCS-like	4aec.1. A	64.55%	0.99	0.88	0.84	35.43%	10.26%	20.20%	34.11%

### Molecular docking

**Fig. 5:**
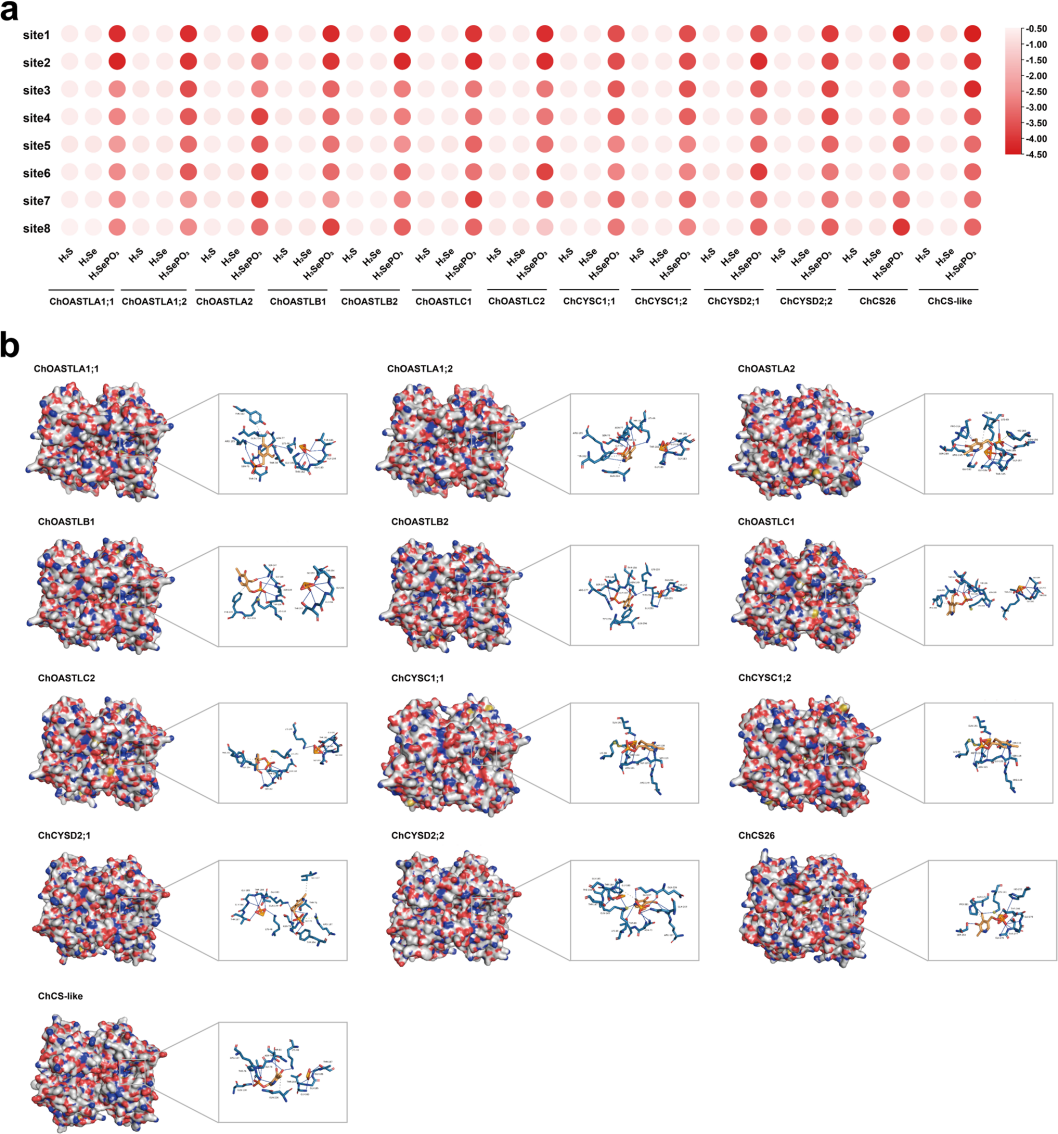
Molecular docking was performed for ChOASTL proteins with ligands (H_2_S, H_2_Se, H_3_SePO_3_) using the Auto Dock Vina program. **a** Heatmap showing binding energies of the different ligands to each binding site of the ChOASTLs (unit: kcal mol^−1^). **b** Interactions of the ternary ChOASTL-PLP-OAS complex with selenophosphate, simulating the binding form of ChOASTL proteins to selenophosphate at the catalytic site. The *left panel* shows the overall picture and the right panel shows the details. Protein is shown on the surface, the amino acid residues at the binding site are shown in *blue*–*green* and the ligands (PLP, OAS, and selenophosphate) are shown in *dark yellow*. The *grey dotted line* represents hydrophobic interactions, the *solid blue line* represents hydrogen bonds, and the *dashed yellow line* represents salt bridges (color figure online)

The members of the ChOASTL protein were predicted with eight potential binding pockets and numbered from highest to lowest probability score of the pocket using the Prankweb online server. Molecular docking was performed for the potential binding pocket of each ChOASTL using the Auto Dock Vina program, and the protein–ligand docking binding energy was recorded. PyMOL was used to export the protein–ligand complexes by PDB files, which were imported into PILP (protein–ligand interaction profiler) for visualization and to analyze the protein–ligand complexes (Adasme et al. 2021; Seeliger and de Groot 2010). Sec or cysteine can be synthesized by enzymatic reduction steps of selenite or sulfite, which is first reduced to selenide or sulfide by sulfite reductase (SiR), followed by *O*-acetyl serine (thiol) lyase catalyzing the reaction of selenide or sulfide with OAS to form Sec or cysteine (Pilon-Smits 2012). Therefore, the sulfide or selenide of substrates was used as ligands for molecular docking with ChOASTL. The results showed that the interaction energy between sulfide or selenide and ChOASTLs ranged from −0.8 to −0.5 kcal mol^−1^, which may indicate that sulfide or selenide compounds experience difficulty when binding to ChOASTL molecules (Fig. 5a, Table S3). Selenide is phosphorylated to selenophosphate by selenophosphate synthetase 2 (SEPHS2) with the participation of ATP and incorporated into the synthesis pathway of Sec (Saito 2021). When selenophosphate was used as a ligand for molecular docking, satisfactory results were obtained, showing that the binding energy was between −4.4 and −2.3 kcal mol^−1^. Thus, selenophosphate may be a potential substrate for interaction with ChOASTL protein molecules.

The web of PLIP was used to process visualized protein–ligand complexes and gain insight into the protein–ligand binding mode constructed by hydrogen bonds, salt bridges, and hydrophobic interactions from amino acid residues, substrates (including selenophosphate and OAS), and cofactor PLP (Fig. 5b). The hydrogen bond is necessary for interactions between the ternary complex ChOASTL-PLP-OAS with selenophosphate. In this study, the highest affinity for substrates was found at two conserved sequences of ChOASTL, which included the PLP binding site TSGNT loop and the catalytic site. At the catalytic site, the ligand was surrounded by Lys^46^, Gly^181^, Thr^182^, Gly^183^, Thr^185^, and Ser^269^, conserved amino acid residues that are binding sites for the cofactor PLP involved in OAS and cysteine synthesis (Hell et al. 2002). The ligands were surrounded by the amino acid residues of the TSGNT loop at the catalytic sites of most ChOASTL and formed hydrogen bonds. However, the ligands were surrounded by one or two amino acid residues of the TSGNT loop in the ChCYSC1;1, ChCYSC1;2, and ChCYSD1;1 proteins. This phenomenon might be explained by the various abilities of ChOASTL family members to bind to selenophosphate at the catalytic site.

### Expression analysis of ChSAT and ChOASTL genes in different tissues under selenium stress

**Fig. 6:**
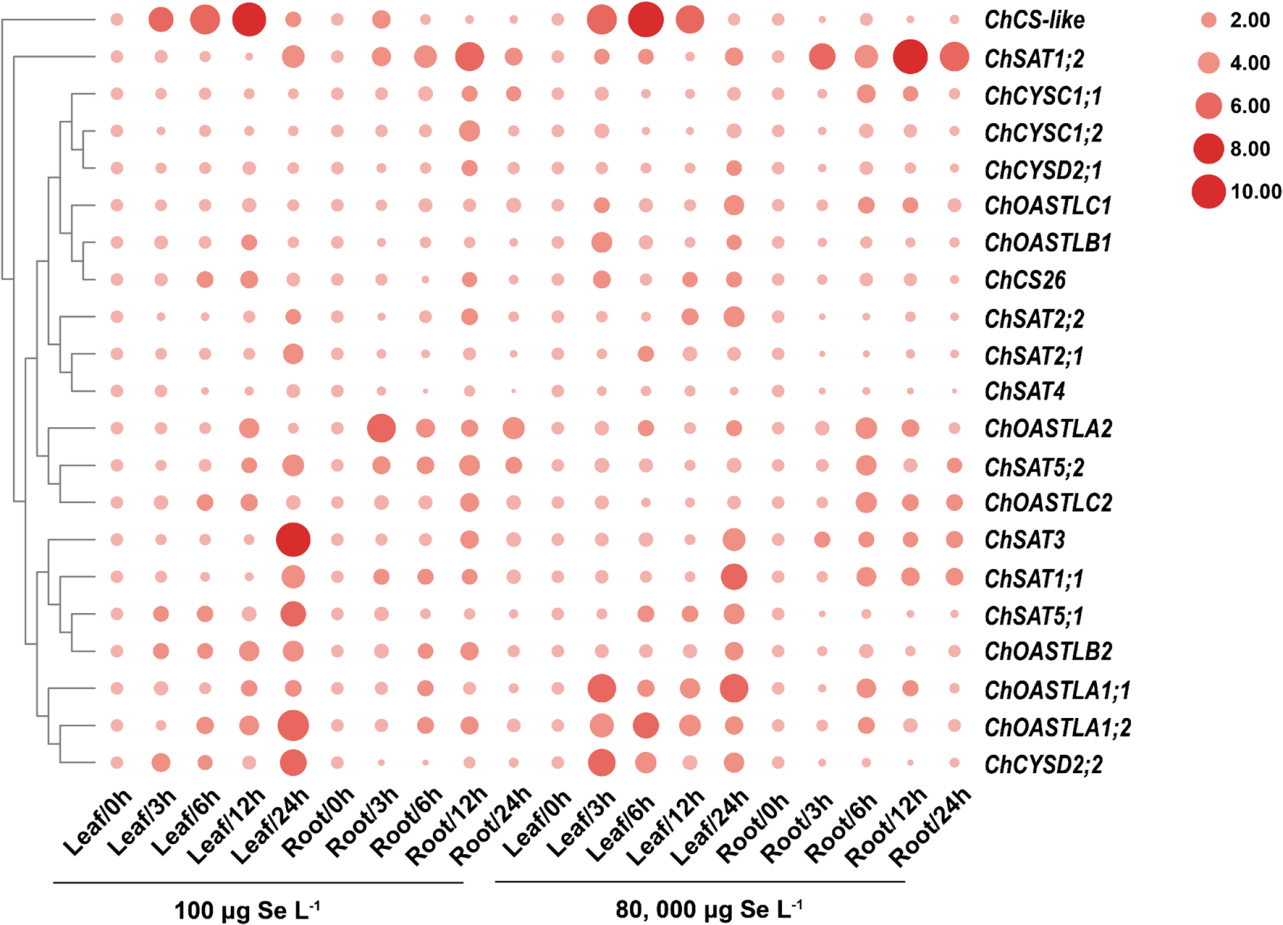
Heatmap showing the expression levels of *ChSAT* and *ChOASTL* genes in leaf and root tissues under different selenium treatments (100 and 80,000 μg Se L^−1^). The *color scale* is indicated in the right part of the heat map (color figure online)

qRT-PCR technology was used to analyze the expression and investigate the response of *ChSAT* and *ChOASTL* genes when the seedlings of *C. hupingshanensis* were treated with selenite. As the seedlings of *C. hupingshanensis* were treated with 100 μg Se L^−1^ selenite reached 24 h, the expression of *ChOASTLA1;2*, *ChCYSD2;2*, and all *ChSAT* genes was significantly upregulated in leaves (Fig. 6). *ChCS-like* was shown to be highly upregulated at 3, 6, 12, and 24 h, with a significant upregulation at 12 h in leaves. In roots, the expression of *ChSAT1;2* and *ChSAT5;2* genes appeared significantly upregulated with increasing duration of selenium stress.

When the seedlings of *C. hupingshanensis* were treated with 80,000 μg Se L^−1^, *ChOASTL1;1*, *ChOASTL1;2*, *ChCYSD2;2*, and *ChCS-like* genes were mainly responsive to high selenium stress, but the expression levels of the *ChSAT* genes did not seem to be significantly different in leaves. In addition, the expression of the *ChSAT1;2* in roots exhibited various upregulations with the duration of high selenium stress, which was more responsive to high selenium stress than the other genes. The upregulation of *ChOASTL* genes in leaves was more obvious than that in roots under selenium stress. Based on these data, the *ChSAT* and *ChOASTL* genes could play an important role in the response to selenium, particularly *ChSAT1;2* and *ChOASTLA1;2*.

### Effect of ChSAT1;2 and ChOASTLA1;2 gene silencing on selenium metabolite content

**Fig. 7:**
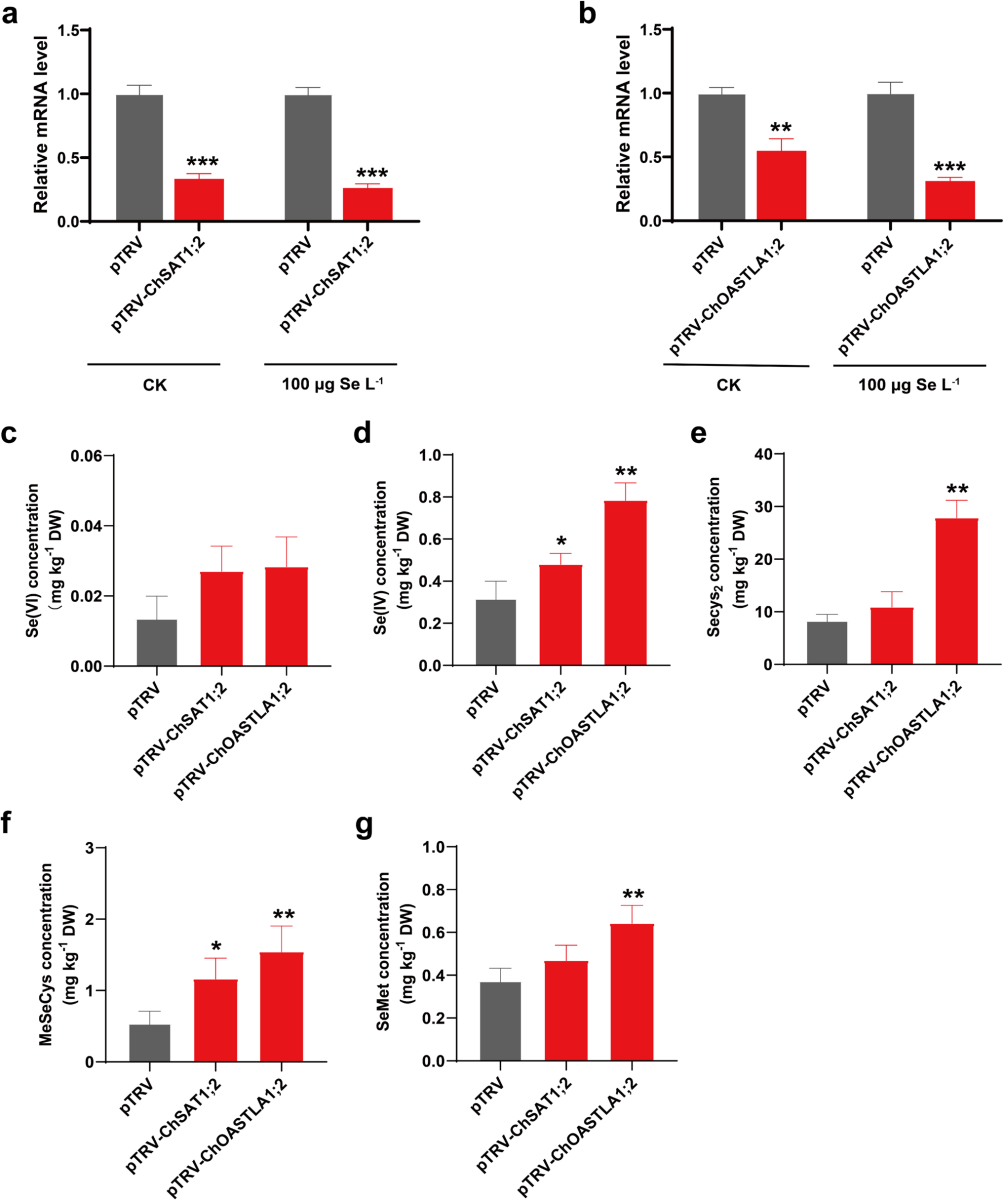
The effect of *ChSAT1;2* and *ChOASTLA1;2* gene silencing on the content of the main selenium metabolites. **a** The expression level of the silenced *ChSAT1;2* genes in *C. hupingshanensis* by untreated (CK) and selenium treatment (100 μg Se L^−1^). **b** The expression level of the silenced *ChOASTLA1;2* genes in *C. hupingshanensis* by untreated (CK) and selenium treatment (100 μg Se L^−1^). The content of Se (VI) **c**, Se (IV) **d**, Secys_2_ **e**, MeSeCys **f**, and MetSe **g** of pTRV, pTRV-*ChSAT1;2*, and pTRV-*ChOASTLA1;2* in *C. hupingshanensis* under selenium treatment (100 μg Se L^−1^) for 24 h. Each data point represents the mean ± standard deviation (SD) (*n* = 3). *Error bars* represent standard deviations. *Asterisks* are used to represent significant differences: *P < 0.05; **P < 0.01; ***P < 0.001

To investigate the important roles of *SAT* and *OASTL* family genes in the selenium metabolic pathway of plants, VIGS was used to investigate the effects of *ChSAT1;2* and *ChOASTLA1;2* gene silencing on the content of five selenium metabolites (Se (VI), Se (IV), SeCys_2_, MeSeCys, SeMet). The 350 bp fragments of *ChSAT1;2* and *ChOASTLA1;2* were cloned into pTRV2, respectively, to obtain pTRV-*ChSAT1;2* and pTRV-*ChOASTLA1;2*. Three weeks after infection with the silencing and control vector, the seedlings of *C. hupingshanensis* were treated with selenite in 100 μg Se L^−1^ for 24 h. qRT-PCR revealed that the expression of *ChSAT1;2* was reduced by more than 60% (Fig. 7a), and the expression of *ChOASTLA1;2* was decreased by about 50% in plants infiltrated with the silencing vectors (Fig. 7b). In addition, compared with the controls, the expression of the *ChSAT1;2* and *ChOASTLA1;2* genes was even more in the *ChSAT1;2* and *ChOASTLA1;2*-silenced plant after selenium treatment. HPLC–ICP-MS analysis indicated that *ChSAT1;2*-silenced plants had elevated accumulations of Se (IV), and MeSeCys, but no significant changes in Se (VI), SeCys_2_, and SeMet levels after selenium treatment compared with controls (Fig. 7e–g). The Se (IV) and MeSeCys content was increased approximately 0.50- and 1.14-fold compared with the negative control. The *ChOASTLA1;2*-silenced plants had significant differences in the content of selenium metabolites, except for Se (VI), after selenium treatment than the control. Surprisingly, the OASTL-catalyzed production of Secys_2_ increased up to 2.32-fold, and the levels of the other three metabolites containing Se (IV), MeSeCys, and SeMet, were increased by 1.46-, 1.84-, and 0.71-fold. The results showed that the silenced *ChSAT1;2* and *ChOASTLA1;2* genes cannot obstruct Sec and other downstream metabolites synthesis in selenium metabolic pathways; on the contrary, it enhances these selenium metabolites synthesis.

## Discussion

In our study, the alignment of members in the *SAT* and *OASTL* families presents conserved functions, and the greater number of members exhibit redundancy. The analysis of conserved motifs and gene structures of the *ChSAT* and* ChOASTL* families showed that the conserved amino acid sequences and gene structures of members in the same subclade were similar (Fig. 3, Fig. S3), which was consistent with the results of *Solanum lycopersicum*, *Sorghum bicolor*, and *Oryza sativa* (Akbudak et al. 2019; Kurt et al. 2021; Liu et al. 2019). However, the number of members in the *SAT* gene family varies significantly between plants as follows: eight *ChSAT* in *C. hupingshanensis,* five *AtSAT* in *Arabidopsis thaliana*, six *OsSAT* in *Oryza sativa,* and six *VvSAT* in *Vitis vinifera* (Tavares et al. 2015). The OASTLs in plants also have multiple isozymes, and the number of members of the *OSATL* gene family also varies between plants (13 *ChOASTL* in *C. hupingshanensis,* 9 *AtOASTL* in *Arabidopsis thaliana*, 9 *OsOASTL* in *Oryza sativa*, 9 *SlOASTL* in *Solanum lycopersicum*, 10 *PtOASTL* in *Populus trichocarpa*, 7 *SbOASTL* in *Sorghum bicolor* and 6 *GmOASTL* in *Glycine max*) (Akbudak et al. 2019; Hatzfeld et al. 2000; Kopriva et al. 2009; Liu et al. 2019; Zhang et al. 2008). Therefore, we speculate that a greater number of *ChSAT* and *ChOASTL* genes were identified in *C. hupingshanensis* than in selenium non-accumulators, which is one of the potential reasons for its ability as a selenium hyperaccumulator to tolerate and accumulate selenium.

The synthesis of Sec is completed by the balance between dissociation and formation of the cysteine synthase complex. The form of the cysteine synthase complex occurred under selenium/sulfur sufficiency conditions, and when the concentration could not satisfy the needs of the plant cell, the complex uncoupled to release free SAT and OASTL (Fig. S7) (Yi et al. 2013). The ten peptides on the C-terminus of SAT occupied the OAS binding site located on the N-terminus of OASTL with the conserved sequence TSGNT (Fig S4, S5), which caused Sec and Cys to unform and trigger the accumulation of OAS. When the cellular OAS concentration reached some point, OAS competed with the binding site, which dissociated the complex, and then the free OASTL-catalyzed Sec and Cys formation. Once the cellular selenium and sulfur concentrations decline to some point, the reaction for the synthesis of Sec and Cys stops, and the cysteine synthase complex remodels. In *A. thaliana*, the formation of the CSC complex was dominated by the interaction of key active site residues Gln^147^, Thr^74^, and Ser^75^ in the TSGNT loop, a conserved motif of the OASTL protein, with the C10 peptide of the SAT protein (Francois et al. 2006). However, the comparison of protein sequences indicates that a mutation in the TSGNT loop of ChOASTL prevents the synthesis of both Sec and Cys (Fig. 4), which leads to an inability to form a complex with SAT.

The concentration of Sec in *C. hupingshanensis *in vivo was regulated by members of the *OASTL* gene family, which could maintain the dynamic balance of the concentration of Sec and may function as a protective mechanism to reduce the toxicity of selenium in plants. In previous studies, the three OASTL isoforms OASTLA1, OASTLB, and OASTLC, which are most highly expressed in *A. thaliana* cells, catalyze the synthesis of cysteine; in contrast, CS-like primarily catalyzes the degradation of cysteine rather than its synthesis and catalyzes the formation of sulfide, ammonia, and pyruvate in a 1:1:1 stoichiometric ratio to maintain the dynamic balance of intracellular cysteine (Alvarez et al. 2010; Heeg et al. 2008; Wirtz and Hell 2006). Due to the chemical similarity of S and Se, the expression of different *OASTL* family genes may regulate the dynamic homeostasis mechanism of the product Sec concentration. In addition, the results of molecular docking suggest that OASTL family proteins exhibit a higher affinity for the ligand selenophosphate; therefore, selenophosphate may be the optimal substrate for the synthesis of Sec (Fig. 5). In our study, the gene members of *ChOASTL* showed a high expression level under selenium stress, especially the expression of *ChOASTLA1;1*, *ChOASTLA1;2*, and *ChCS-like* genes, which were upregulated at high times (Fig. 6). This result possibly suggests that *ChOASTLA*-type and *CS-like* were regulated together by the concentration of Sec in vivo. In the major OASTL isoforms, the highly conserved ^74^TSGNT^78^ loop acts as the active center for cysteine or Sec synthesis by doping with sulfide or selenide (Bonner et al. 2005). Sequence comparisons show that the ^75^Ser of the ^74^TSGNT^78^ loop was replaced by Gly in CS-like (Fig. 4b), which eliminated an important group and caused a change in spatial conformation and function; furthermore, the function of CS-like was changed to degrade Sec or Cys (Hartl et al. 2011). In contrast, the residue responsible for anchoring PLP is highly conserved in all encoded OASTL-like proteins. Analogous to pathways in animal cells, the results should indicate that CS-like participated in regulating the concentration of Sec and Cys in vivo and selenoprotein biosynthesis (Saito 2021).

The levels of Sec and its downstream metabolites depend on the activities and concentration of SAT and OASTL complex, and the expression levels of gene family members. The ChSAT and ChOASTL were encoded by 8 and 13 gene members from *SAT* and *OASTL* families, respectively, which were identified in *C. hupingshanensis.* The SAT and OASTL enzymes are proteins encoded by members of several gene families (Fig. 3, Fig. S3). Although *ChSAT1;2* and *ChOASTLA1;2* were the highest expression genes in their family, which responded to the various concentrations of selenium stress in the seedlings of *C. hupingshanensis*, it cannot be fully demonstrated that Sec synthesis is related only to these two genes. Some studies have demonstrated that knocking out genes in organisms that normally would not cause phenotypic effects is a functional compensation for duplicated genes and that many duplicated genes have remained functionally redundant over long periods of evolutionary time (Hanada et al. 2009). We did not observe a decrease or even a significant increase in Sec content when *ChSAT1;2* and C*hOASTLA1;2* genes were silenced by the VIGS technique. However, other members of the *ChSAT* and *ChOASTL* gene families continued to perform the function of synthesizing Sec instead of them under selenium stress, and even activated stronger levels of Sec, which subsequently influenced the levels of selenium metabolites and selenium metabolism pathways in plants. This indicates that the research on SAT and OASTL genes is crucial for understanding selenium metabolism in plants.

In the future, we will further utilize multi-omics techniques such as genomics, transcriptomic, metabolomic, and epigenomics to comprehensively elucidate the response mechanisms and metabolic regulatory networks of plants to selenium (Guo et al. 2023; Lai et al. 2023; Liu and Zhong 2024), especially delving into the functional mechanisms of SAT and OASTL genes. By analyzing gene expression regulation and methylation modifications, we can better understand the response mechanisms of plants to selenium, providing more insights and methods for future crop improvement, increased selenium content, and enhanced nutritional value.

## Conclusion

In this study, the phylogenetic evolution, chromosome location, gene structure, and expression patterns of the *ChSAT* and *ChOASTL* families were comprehensively analyzed for the first time. Eight *ChSAT* genes and thirteen *ChOASTL* genes were found in *C. hupingshanensis*, which provided a solid basis for exploring the functions of these genes in plant selenium metabolism pathways. In addition, we also explored the affinity of ChOASTL proteins to selenium-containing substrates using molecular docking technology and evaluated the possibility of selenophosphate as a potential substrate for the synthesis of selenocysteine. Furthermore, we also analyzed the expression of *ChSAT* and *ChOASTL* genes under selenium stress in different tissues of *C. hupingshanensis*, and screened out the strongest selenium stress-responsive genes *ChSAT1;2*, and *ChOASTLA1;2*. The silencing of the *ChSAT1;2*, and *ChOASTLA1;2* genes with VIGS technology was verified to affect the levels of selenium metabolites in the plants. The results provide ideas for understanding the functions of the *ChSAT* and *ChOASTL* genes in *C. hupingshanensis*, and provide references for the metabolic pathways of selenium in plants.

### Supplementary Information

Below is the link to the electronic supplementary material.

Supplementary file1 Fig. S1: The metabolic pathway of selenium in plants; Fig. S2: Reaction process of selenocysteine synthesis; Fig. S3: Phylogenetic analysis of the *ChOASTL* families in *C. hupingshanensis*; Fig. S4: Multiplexed alignment of full sequences of SAT protein in *C. hupingshanensis*; Fig. S5: Multiplexed alignment of full sequences of OASTL protein in *C. hupingshanensis*; Fig. S6: The 3D structures of SATs and OASTLs predicted by the SWISS-MODEL; Fig. S7: Formation of the cysteine synthase complex (CSC) to regulate Sec/Cys synthesis (DOCX 4793 KB)

Supplementary file2 Table S1: The coding sequences and protein sequences of *ChSAT* and *ChOASTL* genes; Table S2: The primers of *ChSAT* and *ChOASTL* genes for qRT-PCR and VIGS; Table S3: Molecular docking was performed for ChOASTL proteins with ligands (XLS 99 KB)

## Data Availability

The datasets generated during and analyzed during the current study are available from the corresponding author on reasonable request.
