# Supplementary material for: Gene identification, expression analysis, and molecular docking of SAT and OASTL in the metabolic pathway of selenium in Cardamine hupingshanensis
Source: Plant Cell Rep. 2024 May 22;43(6):148. doi: 10.1007/s00299-024-03227-6 (PMC11111505; doi:10.1007/s00299-024-03227-6)
Supplement: Supplementary file 1 — Supplementary file1 Fig. S1: The metabolic pathway of selenium in plants; Fig. S2: Reaction process of selenocysteine synthesis; Fig. S3: Phylogenetic analysis of the ChOASTL families in C. hupingshanensis; Fig. S4: Multiplexed alignment of full sequences of SAT protein in C. hupingshanensis; Fig. S5: Multiplexed alignment of full sequences of OASTL protein in C. hupingshanensis; Fig. S6: The 3D structures of SATs and OASTLs predicted by the SWISS-MODEL; Fig. S7: Formation of the cysteine synthase complex (CSC) to regulate Sec/Cys synthesis (DOCX 4793 KB) [file 299_2024_3227_MOESM1_ESM.docx]

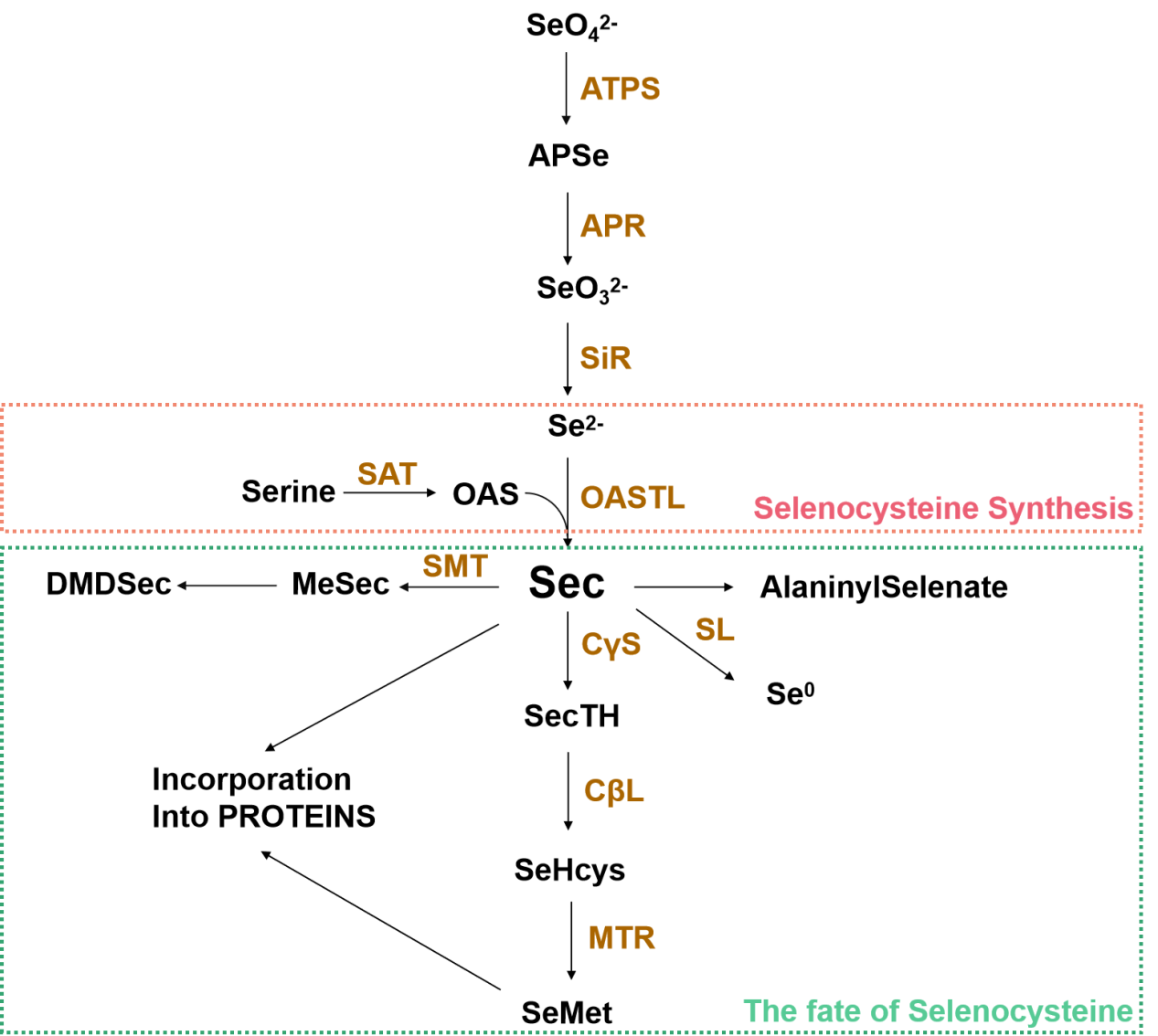


**Fig. S1** The metabolic pathway of selenium in plants. Selenate: SeO_4_^2-^; ATPS: ATP sulfurylase; APSe: adenosine 5'-phosphoselenate; APR: adenosine 5'-phosphosulfate reductase; selenite: SeO_3_^2-^; SiR: sulfite reductase. **Selenocysteine Synthesis:** SAT: serine acetyltransferase; OAS: O-acetylserine; OASTL: O-acetylserine (thiol) lyase; Sec: selenocysteine. **The fate of selenocysteine:** CγS: cystathionine gamma synthase; SecTH: selenocystathionine; CβL: cystathionine beta lyase; SeHcys: selenohomocysteine; MTR: methionine synthase; SeMet: selenomethionine; SMT: selenocysteine methyltransferase; MeSec: methyl-selenocysteine; DMDSe: dimethyl diselenide; SL: selenocysteine lyase.


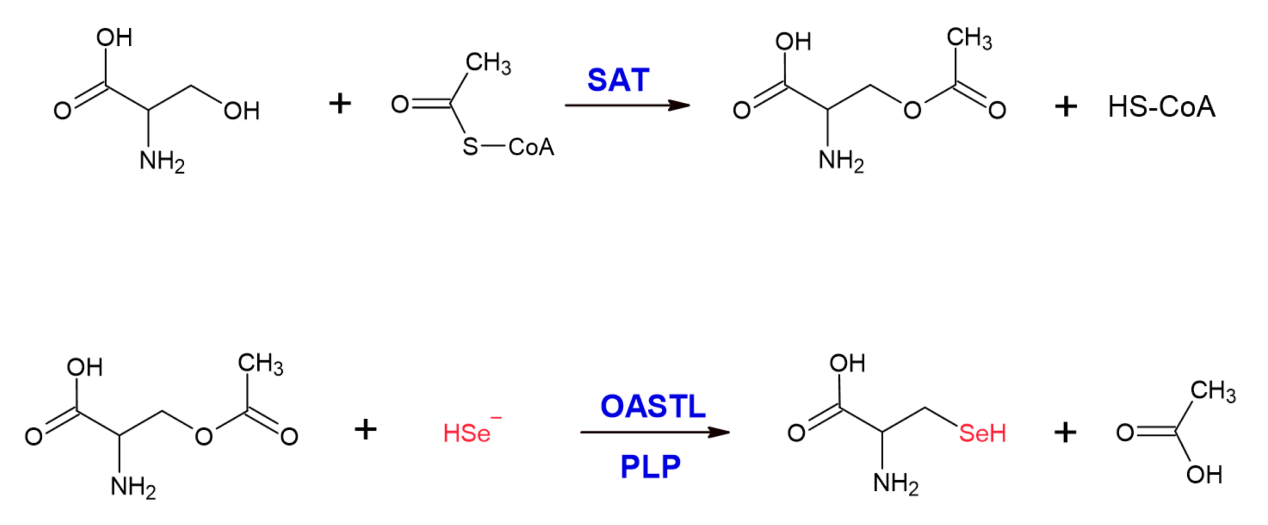


**Fig. S2** Reaction process of selenocysteine synthesis. SAT: serine acetyltransferase; OASTL: O-acetyl serine (thiol) lyase; PLP: pyridoxal 5'-phosphate.


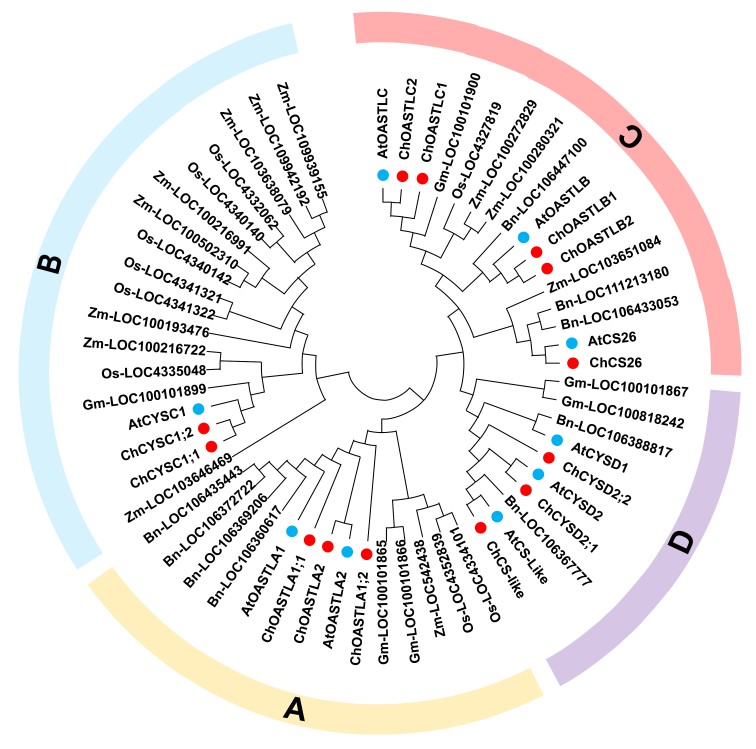


**Fig. S3** Phylogenetic analysis of the *ChOASTL* families in *C. hupingshanensis.* (At: *Arabidopsis thaliana*; Bn: *Brassica napus*; Ch: *Cardamine hupingshanensis*; Gm: *Glycine max*; Os: *Oryza sativa*; Zm: *Zea mays*)


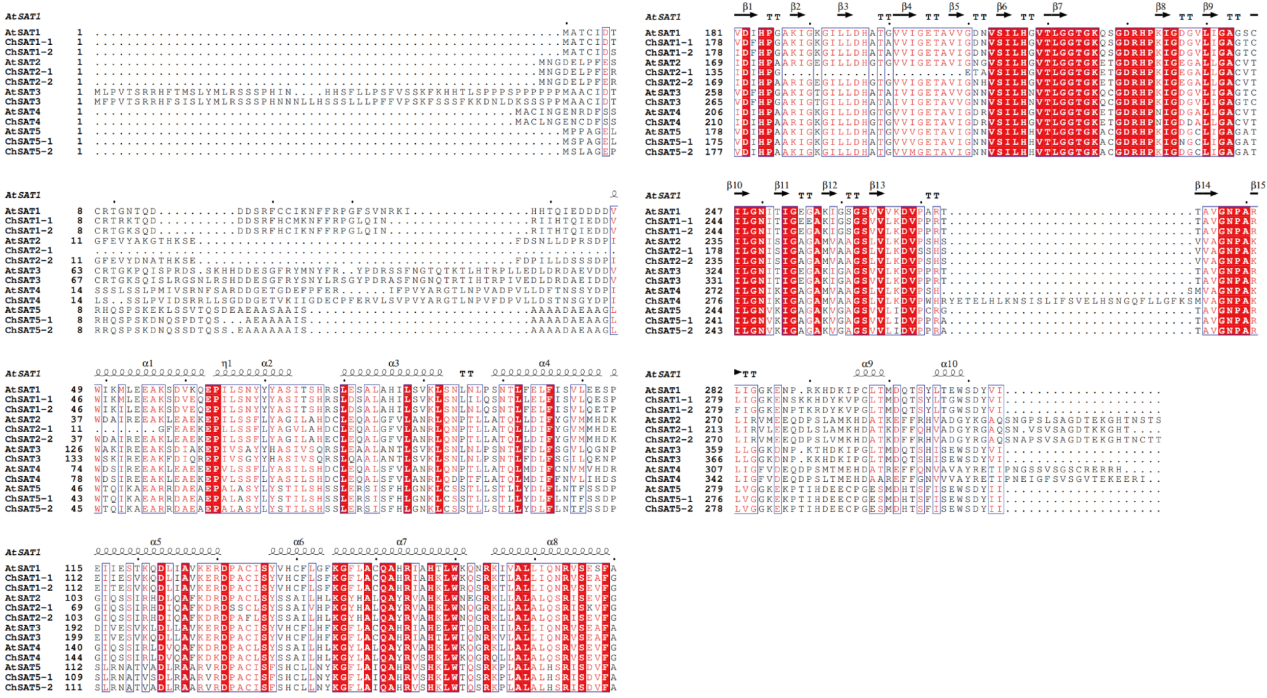


**Fig. S4** Multiplexed alignment of full sequences of SAT protein in *C. hupingshanensis*.


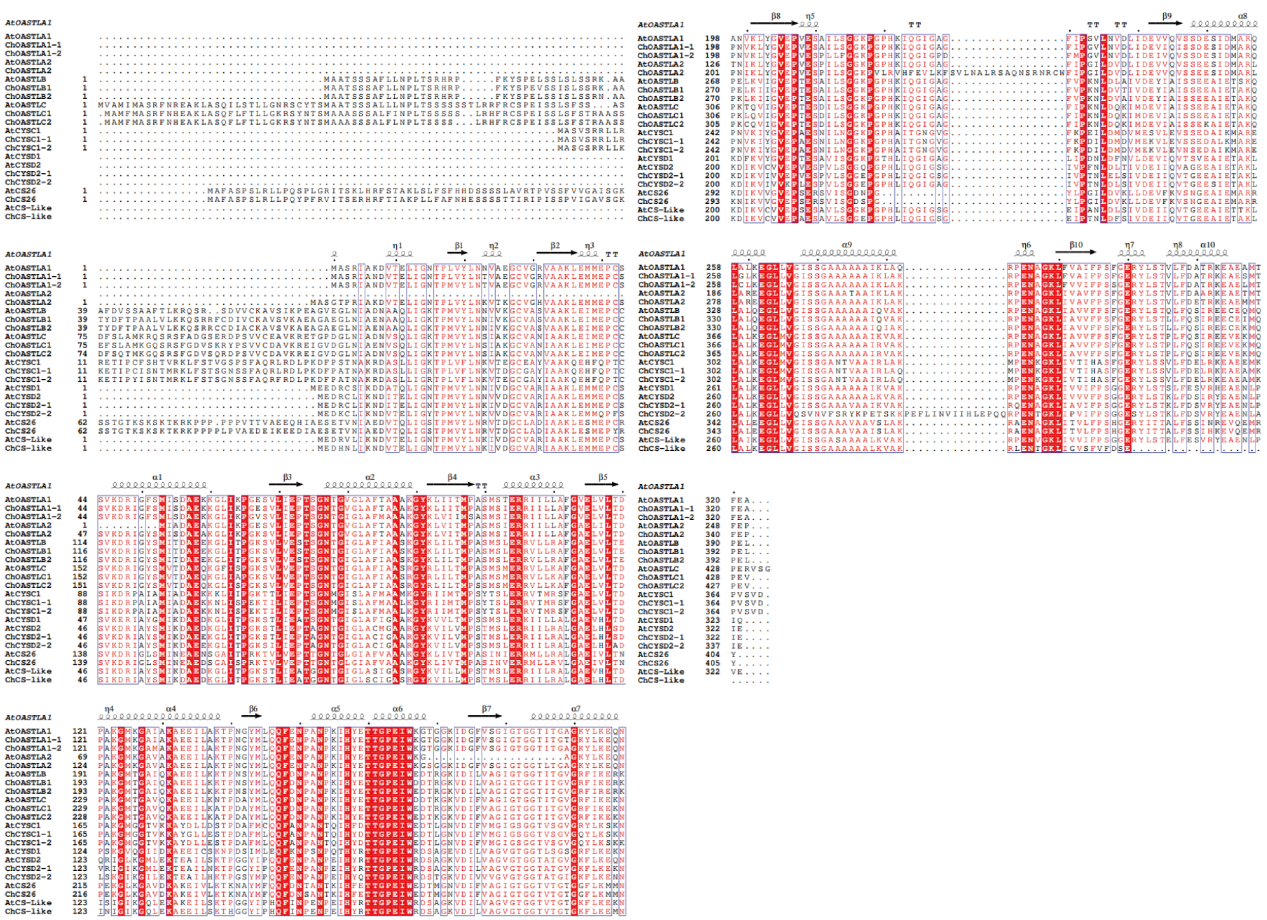


**Fig. S5** Multiplexed alignment of full sequences of OASTL protein in *C. hupingshanensis*.


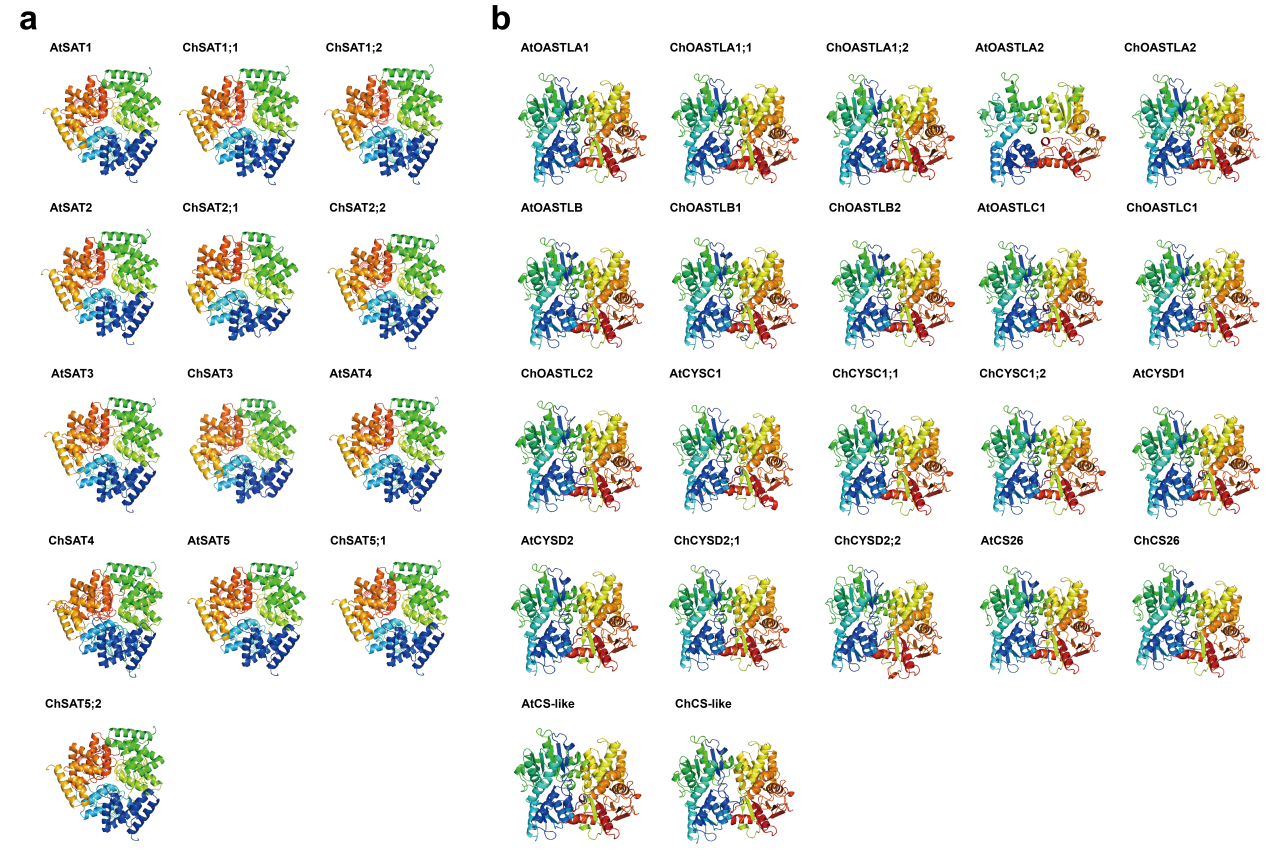


**Fig. S6** The 3D structures of SATs and OASTLs predicted by the SWISS-MODEL. (a) The homo-trimer structures of ChSATs and AtSATs, each monomer is colored shades of blue, green, or red. (b) The homo-dimer structures of ChOASTLs and AtOASTLs, each monomer is colored shades of blue-green or red-yellow.





**Fig. S7** Formation of the cysteine synthase complex (CSC) to regulate Sec/Cys synthesis. Red font represents the active form of SAT and OASTL; black font represents the inactive form of SAT and OASTL.
